# Association of Performance Status With Survival in Patients With Advanced Non–Small Cell Lung Cancer Treated With Pembrolizumab Monotherapy

**DOI:** 10.1001/jamanetworkopen.2020.37120

**Published:** 2021-02-11

**Authors:** Kartik Sehgal, Ritu R. Gill, Page Widick, Poorva Bindal, Danielle C. McDonald, Meghan Shea, Deepa Rangachari, Daniel B. Costa

**Affiliations:** 1 Division of Medical Oncology, Department of Medicine, Beth Israel Deaconess Medical Center, Harvard Medical School, Boston, Massachusetts; 2 Department of Medical Oncology, Dana-Farber Cancer Institute, Boston, Massachusetts; 3 Department of Radiology, Beth Israel Deaconess Medical Center, Harvard Medical School, Boston, Massachusetts

## Abstract

**Importance:**

Despite approximately 40% of patients having Eastern Cooperative Oncology Group (ECOG) performance status (PS) scores of at least 2 in the real world, most landmark clinical trials that led to the use of pembrolizumab as standard of care in advanced non–small cell lung cancer (NSCLC) excluded this group.

**Objective:**

To evaluate whether an ECOG PS score of at least 2 at the start of therapy is associated with progression-free survival (PFS) and overall survival (OS) in advanced NSCLC treated with pembrolizumab monotherapy.

**Design, Setting, and Participants:**

This cohort study included all consecutive patients with advanced NSCLC who underwent treatment with palliative pembrolizumab monotherapy from February 2016 to October 2019 at a single academic cancer center, with data censoring on January 15, 2020.

**Exposures:**

ECOG PS score at start of therapy, with 0 and 1 indicating fully active or restricted in strenuous activity and scores of 2 and higher indicating increasing disability.

**Main Outcomes and Measures:**

PFS and OS, measured from initiation of pembrolizumab monotherapy.

**Results:**

Of 74 patients (median [range] age, 68.5 [33-87] years; 36 [48.7%] women; 53 [71.6%] White individuals) with median follow-up of 19.5 (95% CI, 13.4-27.8) months, 45 (60.8%) had an ECOG PS of 0 or 1, while 29 (39.2%) had an ECOG PS of at least 2. There were no significant differences in the baseline characteristics, except in age. Compared with patients with PS scores of 0 or 1, those with PS scores of at least 2 had significantly lower disease control rates (38 [88.4%] vs 15 [53.6%]; *P* = .002), shorter median PFS (7.9 [95% CI, 4.6-15.4] months vs 2.3 [95% CI, 1.8-4.8] months; *P* = .004), and shorter median OS (23.2 [14.0 vs 35.7] months vs 4.1 [95% CI, 2.1-6.9] months; *P* < .001). Among those potentially eligible for subsequent cancer-directed therapy beyond pembrolizumab monotherapy, patients in the group with PS scores of at least 2 were less likely to receive it than those with PS scores of 0 or 1 (2 [8.3%] vs 14 [45.2%]; *P* = .003). Multivariable adjustment for baseline characteristics confirmed ECOG PS of at least 2 as an independent risk factor for worse PFS (HR, 2.02; 95% CI, 1.09-3.74; *P* = .03) and worse OS (HR, 2.87; 95% CI, 1.40-5.89; *P* = .004).

**Conclusions and Relevance:**

In this cohort study, having an ECOG PS score of at least 2 was associated with poorer prognosis for treatment of advanced NSCLC with palliative pembrolizumab monotherapy. Further prospective studies are needed to evaluate more objective and consistent measures of functional status to facilitate identification of patients with borderline performance status who may achieve durable clinical benefit from treatment with pembrolizumab monotherapy.

## Key Points

**Question:**

Is Eastern Cooperative Oncology Group (ECOG) performance status at the beginning of therapy associated with survival outcomes in patients with advanced non–small cell lung cancer who are treated with palliative pembrolizumab monotherapy?

**Findings:**

In this cohort study of 74 patients, those with ECOG performance status of at least 2 had significantly lower disease control, progression-free survival, and overall survival than those with performance status of 0 or 1. Survival differences remained significant after multivariable adjustment for confounding factors.

**Meaning:**

These findings suggest that ECOG performance status should be considered while making shared therapeutic decisions regarding pembrolizumab monotherapy with patients.

## Teaser

This cohort study evaluates whether an Eastern Cooperative Oncology Group performance status score of at least 2 at the start of therapy is associated with progression-free survival and overall survival in advanced non–small cell lung cancer treated with pembrolizumab monotherapy.

## Introduction

Patients with Eastern Cooperative Oncology Group (ECOG) performance status (PS) scores of 2 or higher compose 34% to 48% of all patients with advanced non–small cell lung cancer (NSCLC).^1,2^ The ECOG PS scale indicates increasing levels of disability, with 0 indicating fully active; 1, restricted in strenuous activity; 2, restricted in work activity but ambulatory and capable of self-care; 3, capable of limited self-care; 4, completely disabled; and 5, dead. Most landmark clinical trials that led to the approval of pembrolizumab and other immune checkpoint inhibitors (ICIs) by the US Food and Drug Administration (FDA) were conducted in patients with ECOG PS scores of 0 or 1.^1,3,4,5^ Due to better perceived tolerance of ICIs compared with cytotoxic chemotherapy, results from these clinical trials have been extrapolated to those with PS scores of 2 or higher, leading to more liberal use of pembrolizumab in this subgroup of patients, who previously might not have been considered eligible for this treatment. This is reflected in the findings from a nationwide patient-level database study that reported an overall increase in the use of systemic therapy at the end of life for patients with NSCLC, driven predominantly by ICIs (along with a concomitant decrease in use of chemotherapy) in the post–ICI approval period.^6^ Moreover, treatment with ICI within 30 days prior to death was associated with decreased referral to hospice and increased in-hospital deaths.^7^ While oncologists routinely use PS for decisions regarding chemotherapy,^8^ rigorous evidence regarding palliative-intent ICI use according to PS is still lacking.

## Methods

### Study Design

We conducted a retrospective cohort study in patients who initiated treatment with palliative-intent pembrolizumab monotherapy for advanced (stage IV or recurrent disease not amenable to curative therapy) NSCLC at an academic cancer center from February 11, 2016, to October 15, 2019, with data censoring on January 15, 2020. This study was conducted in accordance with the research protocols approved by the institutional research board (IRB) of Beth Israel Deaconess Medical Center, the Declaration of Helsinki,^9^ and Good Clinical Practice. Informed consent of individual participants was waived due to no more than minimal risk per the IRB protocol. This study followed the Strengthening the Reporting of Observational Studies in Epidemiology (STROBE) reporting guideline.

### Patients and Procedure

Patients were divided into 2 groups according to ECOG PS scores at the start of pembrolizumab monotherapy: PS of 0 or 1 and PS of 2 or greater. Race was self-reported by participants at the time of registration with the institution. Obesity was defined as body mass index (calculated as weight in kilograms divided by height in meters squared) of at least 30. Creatinine clearance (mL/min/1.73 m^2^ [to convert to milliliter per second per meters squared, multiply by 0.0167]) was calculated using the Cockcroft-Gault equation. Simplified comorbidity score was calculated as described using addition of points for the following parameters: tobacco consumption (ever smoked, 7 points), diabetes treated with either oral hypoglycemics or insulin (5 points), renal insufficiency (creatinine clearance <60 mL/min/1.73 m^2^ according to Cockroft formula, 4 points), respiratory comorbidity (all parameters except the requirement of forced expiratory volume during the first second of <1.5 L, 1 point), cardiovascular comorbidity (1 point), neoplastic comorbidity (1 point), and alcohol use disorder (based on medical record, 1 point).^10^ Regarding sites of metastatic disease, hilar and mediastinal lymph nodes were not included in this category for the purpose of this study. Programmed cell death ligand 1 (PD-L1) tumor proportion score (TPS) in tumor was evaluated using PD-L1 Immunohistochemistry 22C3 pharmDx companion diagnostic test (Agilent). Tumor genomic profile was evaluated using multiplex next-generation sequencing platforms (Foundation One, Foundation One CDx, Foundation ACT [Foundation Medicine], MGH SnapShot [Mass General], and Lung Cancer Mutation Panel [Quest Diagnostics]). For some patients, this was determined using multiple SNaPshot polymerase chain reaction (*KRAS*, *EGFR*, and *BRAF* variants) and fluorescence in-situ hybridization (*ALK* and *ROS1* rearrangement) assays by Integrated Oncology. For those who transferred care to other facilities, living or dead status was obtained through communication with patients or current health care professionals and through publicly available national death databases.

Disease response was independently evaluated for all patients by a thoracic radiologist (R.R.G.) using immune response evaluation criteria in solid tumors (iRECIST). Objective response rate (ORR) included complete response and partial response rates. Disease control rate (DCR) included objective response and stable disease rates. Progression-free survival (PFS) was calculated from the start of pembrolizumab monotherapy until progression of disease, death, or censoring. Progression-free survival 2 (PFS-2) was calculated from the start of pembrolizumab monotherapy until progression of disease on next line of therapy, death, or censoring. Overall survival (OS) was calculated from the start of pembrolizumab monotherapy until death or censoring. Subgroup analyses were performed among patients with ECOG PS scores of at least 2 who received treatment in the following categories: (1) in the first-line setting with any PD-L1 TPS, (2) in any line setting with PD-L1 TPS of at least 50%, (2) in the first-line setting with PD-L1 TPS of at least 50%, and (4) in any line setting with durable clinical benefit (defined as PFS >6 months). Additional analyses were performed by comparing the group with ECOG PS scores of 0 or 1 with a group comprised of patients with ECOG PS scores of 2 only (excluding those with PS 3).

### Statistical Analysis

Categorical data are depicted as numbers and proportions, while continuous data are presented as medians and ranges. Differences between categorical and continuous variables were assessed using 2-sided Fisher exact and Wilcoxon rank-sum tests, respectively. Kaplan-Meier survival and log-rank tests were used to analyze censored survival. Univariate and multivariable Cox proportional hazards regression were performed to examine the association of ECOG PS with PFS and OS. Multivariable adjustments were made for preselected routinely available baseline (ie, at start of pembrolizumab treatment) clinicopathological characteristics that are clinically relevant and/or have been associated with survival outcomes previously in the literature.^10,11,12,13^ These included absolute neutrophil count, number of metastatic sites (surrogate for disease burden), PD-L1 TPS, and simplified comorbidity score for both PFS and OS. Smoking status and age were additionally used in the multivariable regression models for PFS and OS, respectively. The number of variables for multivariable adjustment were chosen taking into account the expected number of patients treated with pembrolizumab monotherapy at our institution. Adjustments for multiple comparisons were not made due to the exploratory nature of this analysis. Stata/IC version 15.1 (StataCorp) and GraphPad version 8.0 (Prism) software were used for graph creation and statistical analysis. Statistical significance was predetermined at a 2-sided *P* < .05.

## Results

In this cohort study of 74 patients (median [range] age, 68.5 [33-87] years; 36 [48.7%] women; 53 [71.6%] White individuals), 29 (39.2%) were alive at median follow up of 19.5 (95% CI, 13.4-27.8) months; 54 [79.2%] received pembrolizumab as a first-line treatment. Overall, 45 patients (60.8%) had ECOG PS scores of 0 or 1 at the start of pembrolizumab monotherapy, while 29 patients (39.2%) had ECOG PS scores of at least 2, including 4 (5.4%) patients with ECOG PS scores of 3 (Table 1). Patients in the group with PS scores of at least 2 were more likely to be older compared with those with PS scores of 0 or 1 (median [range] age, 72 [42-87] years vs 65 [33-87] years; *P* = .003). There were no significant differences between the 2 groups in sex, race, smoking status, simplified comorbidity scores, histology, driver alteration status, number of metastatic sites, or line of therapy; however, patients with ECOG PS scores of at least 2 had numerically higher absolute neutrophil counts as well as higher rates of obesity and PD-L1 TPS of at least 50%. Full details on baseline clinicopathologic and laboratory characteristics are described in Table 1.

**Table 1.** Baseline Clinicopathologic and Laboratory Characteristics

Characteristic	No. (%)			*P* value
	All (N = 74)	ECOG PS score		
		0-1 (n = 45)	≥2 (n = 29)	
ECOG PS				
0	6 (8.1)	6 (13.3)	0	NA
1	39 (52.7)	39 (86.7)	0	
2	25 (33.8)	0	25 (86.2)	
3	4 (5.4)	0	4 (13.8)	
Age, median (range), y	68.5 (33-87)	65 (33-87)	72 (42-87)	.003
Women	36 (48.7)	22 (48.9)	14 (48.3)	>.99
Race				
White	53 (71.6)	32 (71.1)	21 (72.4)	.64
African American	13 (17.6)	7 (15.6)	6 (20.7)	
Asian	8 (10.8)	6 (13.3)	2 (6.9)	
Body mass index, median (range)^a^	24.8 (13.1-47.7)	24.3 (13.1-47.7)	25.1 (17.3-40.9)	.27
Obesity	12 (16.2)	4 (8.9)	8 (27.6)	.05
Ever smoked	66 (89.2)	41 (91.1)	25 (86.2)	.70
Comorbidities				
Simplified comorbidity score, median (range)	9 (0-17)	8 (0-17)	9 (2-14)	.39
Hypertension	36 (48.6)	19 (42.2)	17 (58.6)	.23
Diabetes	10 (13.5)	6 (13.3)	4 (13.8)	>.99
Cardiovascular	42 (56.8)	23 (51.1)	19 (65.5)	.24
Respiratory	23 (31.1)	11 (24.4)	12 (41.14)	.19
Creatinine clearance, mL/min/1.73 m^2^				
<60	26 (35.1)	15 (33.3)	11 (37.9)	.80
<30	3 (4.1)	2 (4.4)	1 (3.4)	>.99
VTE	12 (16.2)	6 (13.3)	6 (20.7)	.52
Histology				
Nonsquamous	52 (70.3)	33 (73.3)	19 (65.5)	.62
Squamous	19 (25.7)	11 (24.4)	8 (27.6)	
Poorly differentiated	3 (4)	1 (2.2)	2 (6.9)	
Driver alterations^b^				
None	29 (42.6)	19 (45.2)	10 (38.5)	.90
* KRAS*	29 (42.6)	17 (4.5)	12 (46.2)	
* EGFR*	4 (5.9)	2 (4.8)	2 (7.7)	
Others	6 (8.8)	4 (9.5)	2 (7.7)	
* ALK*	1 (1.5)	1 (2.4)	0 (0)	
* RET*	1 (1.5)	1 (2.4)	0 (0)	
* ERBB2*	2 (2.9)	2(4.8)	0 (0)	
* MET*	1 (1.5)	0 (0)	1 (3.8)	
* BRAF*	1 (1.5)	0 (0)	1 (3.8)	
PD-L1 TPS, %				
Median (range)	75 (1-100)	80 (2-95)	50 (1-100)	.09
≥50	53 (71.6)	36 (80)	17 (58.6)	.07
Sites of metastases				
Median (range), No.	2 (0-7)	2 (0-7)	2 (1-5)	.14
Lung/pleura	42 (56.8)	25 (55.6)	17 (58.6)	.82
Lymph node	33 (44.6)	18 (40)	15 (51.7)	.35
Brain	25 (33.8)	16 (35.6)	9 (31.0)	.80
Bone	34 (45.9)	18 (40)	16 (55.2)	.24
Liver	10 (13.5)	6 (13.3)	4 (13.8)	>.99
Adrenal	9 (12.2)	5 (11.1)	4 (13.8)	.73
Others	14 (18.9)	6 (13.3)	8 (27.6)	.14
Line of therapy				
First	54 (72.9)	33 (73.3)	21 (72.4)	>.99
Second or greater	20 (27.0)	12 (26.7)	8 (27.6)	
WBC, median (range), /μL	7800 (3300-37 500)	7600 (3300-23 400)	7900 (4700-37 500)	.18
ANC, median (range), /μL	5700 (1900-22 400)	5500 (1900-2300)	6200 (3400-22 400)	.06
Creatinine, median (range), mg/dL	0.9 (0.4-4.2)	0.9 (0.4-4.2)	0.9 (0.4-2.3)	.87

Abbreviations: ANC, absolute neutrophil count; ECOG PS, Eastern Cooperative Oncology Group performance status; PD-L1, programmed cell death ligand 1; TPS, tumor proportion score; VTE, venous thromboembolic disease; WBC, white blood cell count.

SI conversion factors: To convert ANC and WBC to ×10^9^ per microliter, multiply by 0.001; creatinine to micromoles per liter, multiply by 88.4; creatinine clearance rate to milliliter per second per meters squared, multiply by 0.0167.

^a^ Body mass index was calculated as weight in kilograms divided by height in meters squared.

^b^ Driver alterations available for 68 patients (42 [61.8%] with ECOG PS of 1-2; 26 [38.2%] with ECOG PS of ≥2).

**Figure 1.**
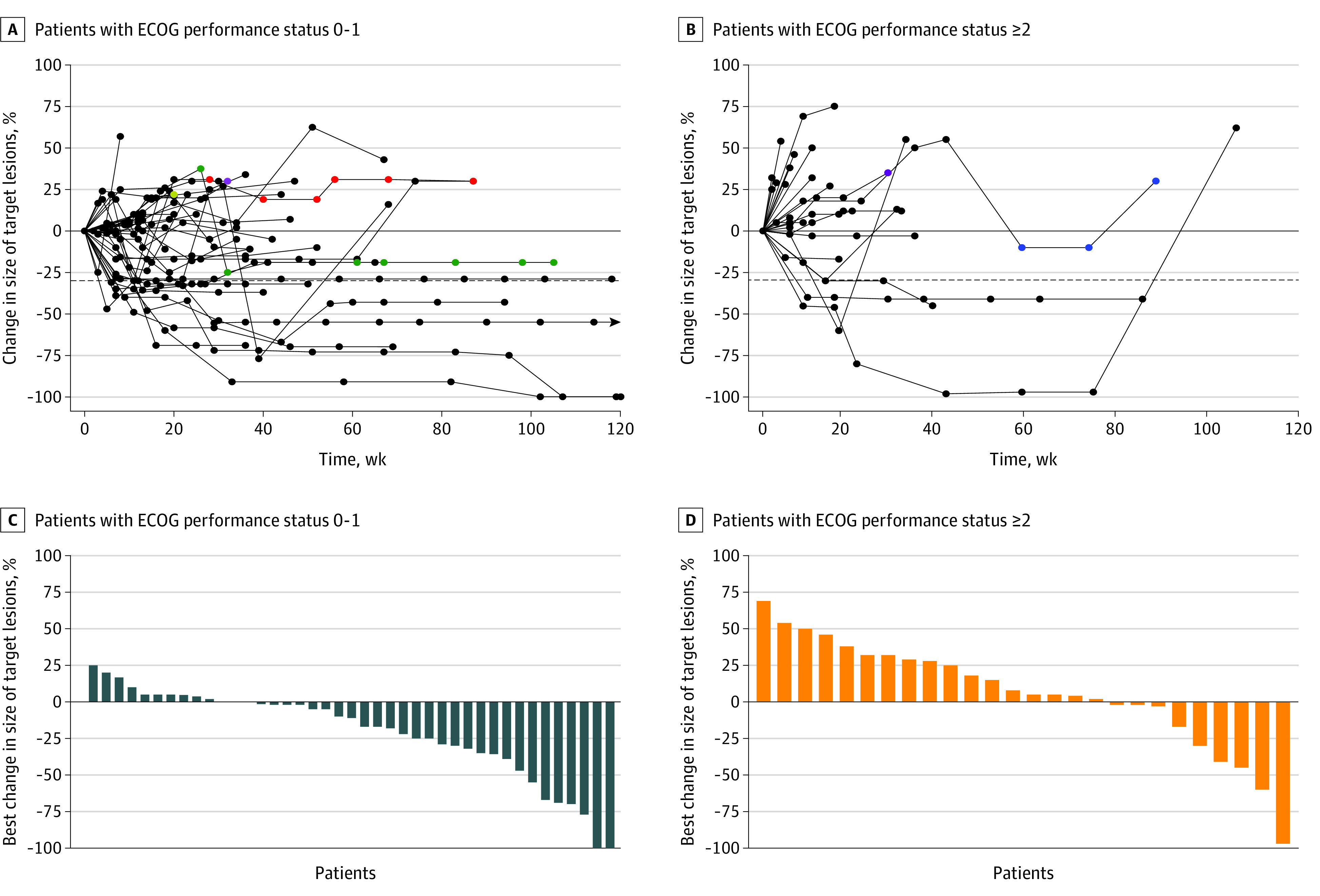
Treatment Response by Performance Status A, B, Black dots represent treatment before confirmed progression of disease. Other dots represent treatment beyond progression, with individual patients represented by unique colors. Arrows represent ongoing treatment. ECOG indicates Eastern Cooperative Oncology Group.

ORR and DCR in the entire cohort were 23.9% (12 of 71) and 74.6% (53 of 71), respectively (Table 2). The group with ECOG PS scores of 0 or 1 had an ORR and a DCR of 27.9% (12 of 43) and 88.4% (38 of 43), respectively, while the group with ECOG PS scores of at least 2 had an ORR and a DCR of 17.9% (5 of 28) and 53.6% (15 of 28), respectively. There were no significant differences between the 2 groups with regards to ORR (*P* = .40). However, patients with ECOG PS scores of at least 2 were less likely to achieve disease control on treatment with pembrolizumab monotherapy (*P* = .002). Spider plots (showing duration of pembrolizumab monotherapy) and waterfall plots (showing the best percentage change in size of target lesions from baseline) for the 2 patient groups are shown in Figure 1. A total of 5 patients (11.1%) with PS scores of 0 or 1 and 2 patients (6.9%) with PS scores of at least 2 were treated beyond progression. Among patients who were potentially eligible for subsequent cancer-directed therapy following pembrolizumab monotherapy, patients in the group with ECOG PS scores of at least 2 were less likely to receive it than those in the group with PS scores of 0 or 1 (2 [8.3%] vs 14 [45.2%]; *P* = .003) (eTable 1 in the Supplement).

**Table 2.** Treatment Outcomes

Outcome	No. (%)			*P* value
	All (N = 74)	ECOG PS score		
		0-1 (n = 45)	≥2 (n = 29)	
Objective response^a^				
ORR	17 (23.9)	12 (27.9)	5 (17.9)	.40
CR	2 (2.8)	2 (4.6)	0	
PR	15 (21.1)	10 (23.3)	5 (17.9)	
Disease control^a^				
DCR	53 (74.6)	38 (88.4)	15 (53.6)	.002
SD	36 (50.7)	26 (60.5)	10 (35.7)	
Immune-related adverse events				
Any grade	33 (44.6)	24 (53.3)	9 (31.0)	.09
Grade ≥3	11 (14.9)	6 (13.3)	5 (17.2)	.74
Steroid use	15 (20.3)	10 (22.2)	5 (17.2)	.77
Treatment discontinuation	7 (9.5)	3 (6.7)	4 (13.8)	.42
Death	1 (1.4)	1 (2.2)	0	>.99
Progression free survival, median, (95% CI), mo	4.8 (2.9-7.9)	7.9 (4.6-15.4)	2.3 (1.8-4.8)	.004
Progression free survival 2, median (95% CI), mo	8.9 (6.6-11.9)	11.9 (8.6-23.9)	4.1 (2.1-6.9)	<.001
Overall survival, median (95% CI), mo	9.9 (6.7-23.2)	23.2 (14.0-35.7)	4.1 (2.1-6.9)	<.001
Death on home hospice				
Yes	29 (39.2)	15 (33.3)	14 (48.3)	.59
No	14 (18.9)	7 (15.6)	7 (24.1)	
Not known	2 (2.7)	0	2 (6.9)	
Not applicable	29 (39.2)	23 (51.1)	6 (20.7)	

Abbreviations: CR, complete response; DCR, disease control rate; ECOG PS, Eastern Cooperative Oncology Group performance status; ORR, overall response rate; PR, partial response; SD, stable disease.

^a^ Objective response and disease control rates available for 71 patients (43 [60.6%] with ECOG PS 0-1; 28 [39.4%] with ECOG PS ≥2).

**Figure 2.**
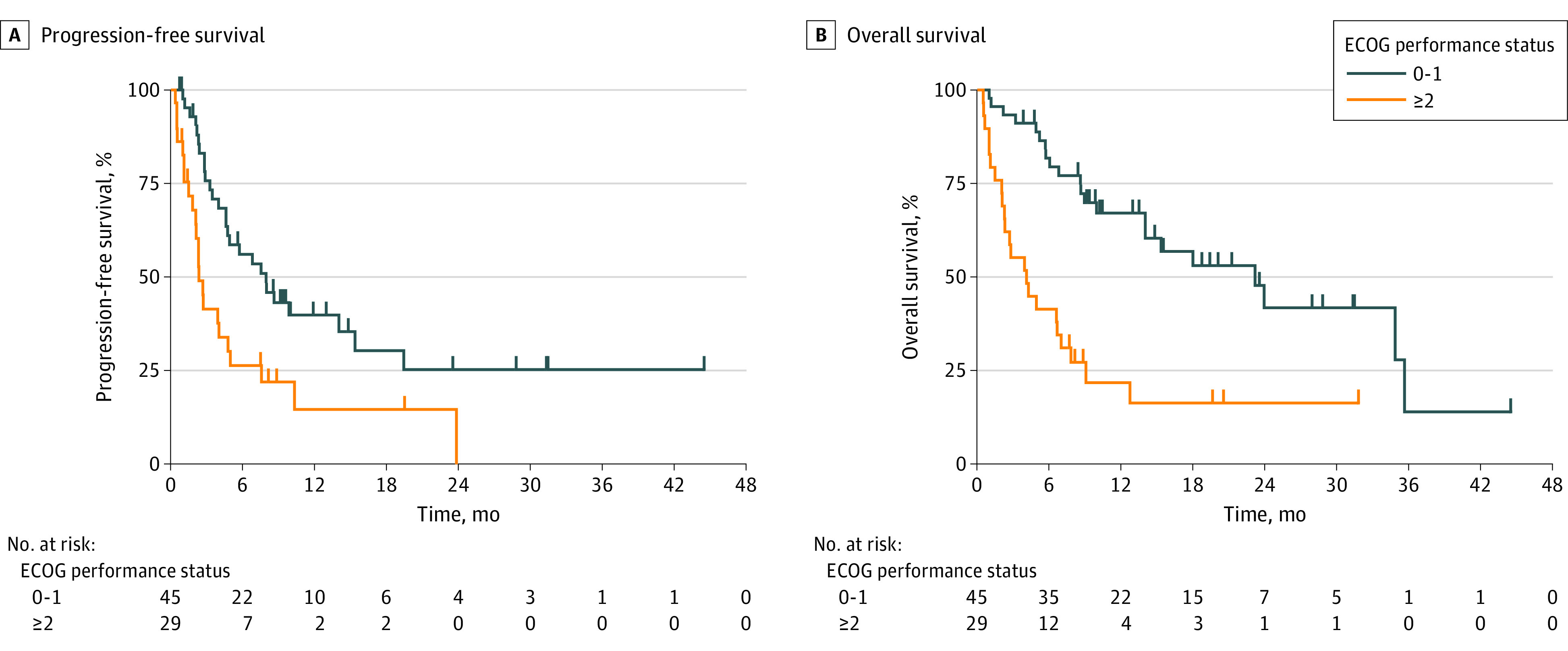
Outcomes by Performance Status ECOG indicates Eastern Cooperative Oncology Group.

Median PFS, PFS-2, and OS in the entire cohort were 4.8 (95% CI, 2.9-7.9) months, 8.9 (95% CI, 6.6-11.9) months, and 9.9 (95% CI, 6.7-23.2) months, respectively. Compared with the group with ECOG PS scores of 0 or 1, the group with ECOG PS scores of at least 2 had significantly shorter median PFS (7.9 [95% CI, 4.6-15.4] months vs 2.3 [95% CI, 1.8-4.8] months; *P* = .004) (Figure 2A and Table 2). Compared with the group with ECOG PS scores of 0 or 1, the group with ECOG PS scores of at least 2 also had significantly shorter median PFS-2 (11.9 [95% CI, 8.6-23.9] months vs 4.1 [95% CI, 2.1-6.9] months; *P* < .001) (Table 2; eFigure 1 in the Supplement). Compared with the group with ECOG PS scores of 0 or 1, patients in the group with ECOG PS scores of at least 2 also had significantly shorter OS (23.2 [95% CI, 14.0-35.7] months vs 4.1 [95% CI, 2.1-6.9] months; *P* < .001) (Figure 2B and Table 2). A greater number of patients had any grade immune-related adverse events in the group with ECOG PS scores of 0 or 1 than in the group with ECOG PS scores of at least 2 (24 [53.3%] vs 9 [31.0%]), but the difference did not reach statistical significance (*P* = .09). There was no difference in the number of patients with grade 3 or higher immune-related adverse events among both groups (PS 0-1, 6 [13.3%] vs PS ≥2, 5 [17.2%]; *P* = .74). Treatment-related deaths occurred in 1 patient (2.2%) in the group with ECOG PS scores of 0 or 1 due to pneumonitis in 2016.

Unadjusted analysis of baseline clinicopathologic and laboratory characteristics at start of pembrolizumab monotherapy showed ECOG PS, white blood cell count, and absolute neutrophil count to be associated with PFS and OS (eTable 2 in the Supplement). Multivariable adjustment for preselected baseline characteristics confirmed ECOG PS score of at least 2 at start of pembrolizumab monotherapy as a significant independent risk factor for worse PFS (hazard ratio [HR] for progression or death, 2.02; 95% CI, 1.09-3.74; *P* = .03) and worse OS (HR for death, 2.87; 95% CI, 1.40-5.89; *P* = .004) (Table 3). Higher absolute neutrophil count also remained independently associated with worse PFS and OS in this analysis.

**Table 3.** Adjusted Association Between Baseline Clinicopathologic and Laboratory Characteristics and Survival Outcomes

Characteristic	Progression or death, adjusted HR (95% CI)	*P* value	Death, adjusted HR (95% CI)	*P* value
ECOG PS, ≥2 vs 0-1	2.02 (1.09-3.74)	.03	2.87 (1.40-5.89)	.004
Absolute neutrophil count	1.08 (1.01-1.16)	.02	1.12 (1.04-1.22)	.004
Metastatic sites, No.	1.18 (0.95-1.48)	.14	1.15 (0.88-1.49)	.29
PD-L1 TPS, %	0.99 (0.99-1.01)	.70	1.00 (0.99-1.01)	.99
Simplified comorbidity score	1.03 (0.91-1.15)	.66	0.99 (0.88-1.11)	.87
Ever smoker, no vs yes	4.71 (1.15-19.29)	.03	NA	NA
Age, y	NA	NA	1.02 (0.99-1.01)	.99

Abbreviations: ECOG PS, Eastern Cooperative Oncology Group performance status; HR, hazard ratio; NA, not applicable; PD-L1, programmed cell death ligand 1; TPS, tumor proportion score.

Subgroup analyses in patients with an ECOG PS score at least 2 and tumor PD-L1 TPS of at least 50%, any tumor PD-L1 TPS treated with pembrolizumab in the first-line setting, and tumor PD-L1 TPS of at least 50% who were treated with pembrolizumab in the first-line setting showed results consistent with prior studies, with the major exception of shorter OS in our cohort compared with the Pembrolizumab in Patients With Non–Small Cell Lung Cancer of Performance Status 2 (PePS2) trial^7,14,15,16,17^ (eTable 3 in the Supplement). Further analysis of patients belonging to the group with ECOG PS scores of at least 2 who achieved vs did not achieve durable clinical benefit (defined as PFS >6 months) revealed no differences in baseline clinicopathological characteristics (eTable 4 in the Supplement); however, those with durable clinical benefit had a higher likelihood of developing any grade immune-related adverse events than those without durable clinical benefit (5 [71.4%] vs 4 [18.2%]; *P* = .02). Additional comparison of patients with ECOG PS scores of 0 or 1 with patients with ECOG PS scores of 2 only (excluding 4 patients [13.8%] with PS 3) at the beginning of pembrolizumab monotherapy showed results consistent with aforementioned findings (eFigure 2 and eTable 5 in the Supplement).

## Discussion

Our study reported a 39.2% prevalence rate of ECOG PS scores of at least 2 in patients with advanced NSCLC at the start of pembrolizumab monotherapy (including first-line therapy in 54 patients [79.2%]), which is consistent with reports from other colleagues in the real-world setting.^1,2^ In addition, the median PFS and OS of 7.9 and 23.2 months, respectively, in the group with ECOG PS scores of 0 to 1 is consistent with those reported in the registrational KEYNOTE-024 trial of previously untreated patients only.^4^ The major finding from our study is the association of ECOG PS scores of 2 or greater at the start of therapy with worse survival in patients with advanced NSCLC treated with palliative-intent pembrolizumab monotherapy. To account for the retrospective nature of analysis, we adjusted these results for routinely available and clinically relevant baseline clinicopathological features at the start of pembrolizumab treatment. These included clinical (age, smoking status, and comorbidities), laboratory (absolute neutrophil count), imaging (disease burden), and pathologic (PD-L1 TPS) characteristics. Of these, PD-L1 TPS is an FDA-approved biomarker for use of ICIs.^11^ Simplified comorbidity score, an alternative for Charlson Comorbidity Index, was chosen to adjust for the association of other comorbidities with survival outcomes.^10^ Despite limitations related to nonadjustment for other potential confounding variables, such as obesity and tumor genomic alterations (eg, *EGFR*-altered NSCLC), our results merit close consideration in light of increasing use of ICIs in the real-world among patients who might not otherwise be considered treatment eligible.

Our findings are supported by other recently published retrospective reports also demonstrating a negative prognostic association of PS scores of at least 2 with OS in patients treated with ICIs for advanced NSCLC. Petrillo et al^7^ reported a median OS of 4.5 months in a similar cohort of patients with ECOG PS scores of at least 2, although their study included patients treated with nivolumab, pembrolizumab, or atezolizumab. Other retrospective studies have focused on a further selected subgroup of patients with advanced NSCLC with PD-L1 TPS of at least 50% and ECOG PS scores of 2 treated with first-line pembrolizumab monotherapy.^14,15,16^ Results from the subgroup analysis in our cohort mirror the results reported from the largest study of such patients,^14^ which reported a median PFS and OS of 2.4 months and 3.0 months, respectively (eTable 3 in the Supplement).

PePS2 is the only prospective, single-arm phase 2 trial^17^ that has evaluated pembrolizumab monotherapy exclusively in patients with NSCLC and ECOG PS scores of 2. An ORR of 27% (95% CI, 17%-39%), DCR of 37% (95% CI, 26%-49%), and a drug toxic effect rate of 28% (95% CI, 19%-41%) were observed. A median OS of 9.8 (95% CI, 7.1-14.6) months was reported as a secondary outcome in the entire cohort. This is substantially longer than the median OS of 4.1 months and 4.5 months reported by our cohort and by Petrillo et al,^7^ respectively (eTable 3 in the Supplement). CheckMate 153, a phase 3b/phase 4 study of nivolumab in previously treated patients with advanced NSCLC^18^ reported a median OS of 4.0 months in the subgroup of patients with ECOG PS scores of 2. The rates of any grade treatment-related adverse events (TRAEs) and grade 3 or 4 TRAEs were 29% and 9%, respectively.^18^ These are in consonance with our findings. In those with PD-L1 TPS of at least 50% and irrespective of which line of therapy, median OS was 14.6 months (95% CI, 4.6 months-not reached) in the PePS2 trial,^17^ again, much longer than the median OS of 2.8 months in our cohort. Ongoing prospective studies (NCT03351361 and NCT02869789) appraising the impact of ICI treatment on overall survival in patients with NSCLC with PS scores of 2 and higher are crucial to further evaluate this patient population.

### Limitations

The limitations of this study include small sample size, inclusion of a few patients with ECOG PS scores of 3, treatment at a single academic center, and unmeasured biases associated with the retrospective nature of our analysis. Patients with ECOG PS scores of at least 2 were significantly older, had numerically but not statistically significant higher absolute neutrophil counts, and had higher rates of obesity and PD-L1 TPS of at least 50%. While we included most of these clinicopathological characteristics in our multivariable model, adjustments were not made for obesity status and individual tumor genomic alterations. Other patient characteristics that were not available for this analysis and are potentially relevant include absolute lymphocyte count, tumor mutational burden, status of chronic viral infections (eg, HIV, hepatitis B, and hepatitis C), mental health comorbidities, geriatric assessment measures, status of activities of daily living as well as independent activities of daily living, and social determinants of health, such as education status, housing status, and income level. In addition, most patients were White individuals; therefore, these results may not be applicable to other patient populations. Finally, interoperator variability in assignation of performance status (by 4 thoracic medical oncologists [M.S., D.R., D.B.C., and another researcher]) may have affected the results of this study.^2,19^

## Conclusions

Despite these limitations, our results closely align with those from multiple retrospective reports, showing that a ECOG PS score of at least 2 was associated with poorer prognosis. These findings underscore the importance of further evaluation of the effect of PS on survival outcomes in patients with advanced cancer treated with ICIs in palliative-intent settings. Despite recent technological advances and a new standard of care in ICIs as first-line therapy in those without targetable genomic alterations, therapeutic decisions for patients with moderate to poor performance status remain akin to walking on a tightrope to avoid undertreatment of patients likely to benefit while minimizing avoidable harm.^19^ It is imperative to include more objective, consistent, and dynamic measurements of functional status in future clinical trials to facilitate identification of those with borderline PS who could achieve durable clinical benefit and improvement in quality of life from treatment with ICIs.^20^

## Notes

Supplement. **eTable 1. **Next-Line Therapies **eTable 2. **Unadjusted Association Between Baseline Clinicopathologic/Laboratory Characteristics and Patient Outcomes **eTable 3. **Comparison of Efficacy and Survival Outcomes Reported in Literature on Treatment With Immune Checkpoint Inhibitors in Patients With Advanced NSCLC with ECOG PS of at Least 2 **eTable 4. **Clinicopathologic and Treatment Characteristics of Patients With ECOG PS of at Least 2 With or Without Durable Clinical Benefit **eTable 5. **Treatment Outcomes in Patients With ECOG PS 0-1 vs ECOG PS2 **eFigure 1. **Univariate Kaplan-Meier Survival Curves for Progression-Free Survival-2 in Patients With Advanced NSCLC and ECOG PS 0-1 vs at Least 2 Treated With Pembrolizumab Monotherapy **eFigure 2. **Univariate Kaplan-Meier Survival Curves for Progression-Free Survival, Overall Survival, and Progression-Free Survival in Patients With Advanced NSCLC and ECOG PS 0-1 vs 2 Treated With Pembrolizumab Monotherapy
