# Supplementary material for: Association of Performance Status With Survival in Patients With Advanced Non–Small Cell Lung Cancer Treated With Pembrolizumab Monotherapy
Source: JAMA Netw Open. 2021 Feb 11;4(2):e2037120. doi: 10.1001/jamanetworkopen.2020.37120 (PMC7879233; doi:10.1001/jamanetworkopen.2020.37120)
Supplement: Supplement. — eTable 1. Next-Line Therapies eTable 2. Unadjusted Association Between Baseline Clinicopathologic/Laboratory Characteristics and Patient Outcomes eTable 3. Comparison of Efficacy and Survival Outcomes Reported in Literature on Treatment With Immune Checkpoint Inhibitors in Patients With Advanced NSCLC with ECOG PS of at Least 2 eTable 4. Clinicopathologic and Treatment Characteristics of Patients With ECOG PS of at Least 2 With or Without Durable Clinical Benefit eTable 5. Treatment Outcomes in Patients With ECOG PS 0-1 vs ECOG PS2 eFigure 1. Univariate Kaplan-Meier Survival Curves for Progression-Free Survival-2 in Patients With Advanced NSCLC and ECOG PS 0-1 vs at Least 2 Treated With Pembrolizumab Monotherapy eFigure 2. Univariate Kaplan-Meier Survival Curves for Progression-Free Survival, Overall Survival, and Progression-Free Survival in Patients With Advanced NSCLC and ECOG PS 0-1 vs 2 Treated With Pembrolizumab Monotherapy [file jamanetwopen-e2037120-s001.pdf]

Supplemental Online Content

Sehgal K, Gill RR, Widick P, et al. Association of performance status with survival in patients with advanced non–small cell lung cancer treated with pembrolizumab monotherapy. *JAMA Netw Open*. 2021;4(2):e2037120.
doi:10.1001/jamanetworkopen.2020.37120

eTable 1. Next-Line Therapies
eTable 2. Unadjusted Association Between Baseline Clinicopathologic/Laboratory Characteristics and Patient Outcomes
eTable 3. Comparison of Efficacy and Survival Outcomes Reported in Literature on Treatment With Immune Checkpoint Inhibitors in Patients With Advanced NSCLC with ECOG PS of at Least 2
eTable 4. Clinicopathologic and Treatment Characteristics of Patients With ECOG PS of at Least 2 With or Without Durable Clinical Benefit
eTable 5. Treatment Outcomes in Patients With ECOG PS 0-1 vs ECOG PS2
eFigure 1. Univariate Kaplan-Meier Survival Curves for Progression-Free Survival-2 in Patients With Advanced NSCLC and ECOG PS 0-1 vs at Least 2 Treated With Pembrolizumab Monotherapy
eFigure 2. Univariate Kaplan-Meier Survival Curves for Progression-Free Survival, Overall Survival, and Progression-Free Survival in Patients With Advanced NSCLC and ECOG PS 0-1 vs 2 Treated With Pembrolizumab Monotherapy

This supplemental material has been provided by the authors to give readers additional information about their work.

© 2021 Sehgal K et al. *JAMA Network Open.*

eTable 1. Next-Line Therapies

Agents	Regimen	ECOG PS 0-1 (N = 31)	ECOG PS ≥2 (N = 24)
**Pembrolizumab + Chemotherapy**	Pembrolizumab + Carboplatin + Pemetrexed	3	
	Pembrolizumab + Carboplatin + Paclitaxel	1	
	Pembrolizumab + Pemetrexed	1	
**Chemotherapy**	Carboplatin + Pemetrexed	2	
	Carboplatin + Paclitaxel	1	
	Carboplatin + Gemcitabine	1	
	Docetaxel	2	
**Targeted therapy**	Osimertinib	1	1
	Alectinib	1	
	Dabrafenib + Trametinib		1
**Clinical Trials**		1	
**Total**		**14**	**2**

© 2021 Sehgal K et al. *JAMA Network Open.*

eTable 2. Unadjusted Association Between Baseline Clinicopathologic/Laboratory Characteristics and Patient Outcomes

	**Unadjusted HR for progression or death (95% CI)**	**P value**	**Unadjusted HR for death (95% CI)**	**P value**
ECOG PS (2-3 versus 0-1)	**2.24 (1.28 – 3.92)**	**0.005**	**3.59 (1.94 – 6.65)**	**<0.001**
Age (years	1.01 (0.98 – 1.04)	0.42	1.03 (0.99 – 1.06)	0.09
Sex (Female versus Male)	1.65 (0.94 – 2.89)	0.08	1.39 (0.78 – 2.49)	0.27
Race (Caucasian versus Non-Caucasian)	0.79 (0.42 – 1.54)	0.50	0.63 (0.31 – 1.27)	0.19
Body mass index	1.01 (0.97 – 1.05)	0.66	1.00 (0.95 – 1.05)	0.96
Obese (Yes versus No)	1.36 (0.69 – 2.65)	0.38	1.44 (0.69 – 3.02)	0.33
Ever smoker, (No versus Yes)	2.84 (0.99 – 8.20)	0.05	1.76 (0.74 – 4.19)	0.20
Simplified Comorbidity score	0.98 (0.89 – 1.08)	0.67	1.03 (0.94 – 1.14)	0.48
Hypertension (Yes versus No)	1.08 (0.62 – 1.88)	0.79	1.35 (0.75 – 2.43)	0.32
Diabetes mellitus (Yes versus No)	1.24 (0.52 – 2.94)	0.62	1.69 (0.78 – 3.68)	0.18
Cardiovascular Comorbidities (Yes versus No)	0.93 (0.53 – 1.63)	0.80	1.25 (0.69 – 2.27)	0.46
Respiratory Comorbidities (Yes versus No)	0.89 (0.49 – 1.59)	0.69	1.10 (0.58 – 2.09)	0.76
Creatinine clearance <60 (Yes versus No)	1.04 (0.58 – 1.85)	0.90	1.36 (0.74 – 2.47)	0.32
Creatinine clearance <30 (Yes versus No)	1.52 (0.47 – 4.94)	0.48	2.64 (0.81 – 8.61)	0.11
VTE (Yes versus No)	0.85 (0.41 – 1.76)	0.67	0.87 (0.39 – 1.91)	0.73
PD-L1 TPS (%)	0.99 (0.99 – 1.01)	0.77	0.99 (0.99 – 1.01)	0.51
Number of metastatic sites	1.14 (0.93 – 1.39)	0.19	1.12 (0.90 – 1.38)	0.31
Lung/pleura metastases (Yes versus No)	0.79 (0.45 – 1.39)	0.42	0.72 (0.39 – 1.30)	0.27
Lymph node metastases (Yes versus No)	1.25 (0.71 – 2.17)	0.44	1.09 (0.59 – 1.99)	0.78
Brain metastases (Yes versus No)	1.05 (0.58 – 1.89)	0.87	1.15 (0.61 – 2.15)	0.67
Bone metastases (Yes versus No)	1.61 (0.92 – 2.83)	0.09	1.41 (0.78 – 2.56)	0.26
Liver metastases (Yes versus No)	1.81 (0.76 – 4.32)	0.18	1.54 (0.68 – 3.49)	0.29

© 2021 Sehgal K et al. *JAMA Network Open.*

Adrenal metastases (Yes versus No)	1.26 (0.54 – 2.98)	0.59	1.67 (0.70 – 3.97)	0.25
Other metastases (Yes versus No)	1.39 (0.65 – 2.97)	0.39	1.44 (0.71 – 2.91)	0.32
Line of therapy (≥ Second line versus First line)	1.02 (0.55 – 1.89)	0.95	0.97 (0.49 – 1.88)	0.92
White blood cell count (K/uL)	**1.06 (1.01 – 1.12)**	**0.01**	**1.08 (1.03 – 1.13)**	**0.003**
Absolute neutrophil count (K/uL)	**1.07 (1.01 – 1.14)**	**0.02**	**1.09 (1.03 – 1.17)**	**0.004**
Creatinine (mg/dL)	0.94 (0.53 – 1.68)	0.84	1.22 (0.68 – 2.18)	0.66

Abbreviations: CAD = Coronary artery disease, CHF = Congestive heart failure, CI = Confidence intervals, ECOG PS = Eastern Cooperative Oncology group performance status, HR = Hazard ratio, K/uL = x1000/microliter, NSCLC = Non-small-cell lung cancer, PD-L1 = Programmed death ligand -1, TPS = Tumor proportion score, VTE = Venous thromboembolic disease

© 2021 Sehgal K et al. *JAMA Network Open.*

eTable 3. Comparison of Efficacy and Survival Outcomes Reported in Literature on Treatment With Immune Checkpoint Inhibitors in Patients With Advanced NSCLC with ECOG PS of at Least 2

	Retrospective					Prospective Non-randomized
	**Sehgal *et al***	**Petrillo *et al***	**Facchinetti *et al***	**Alessi *et al***	**Friedlaender *et al***	**Middleton *et al***
**ICI**	Pembrolizumab	Nivolumab, Pembrolizumab, Atezolizumab	Pembrolizumab	Pembrolizumab	Pembrolizumab	Pembrolizumab
**PS**	2 - 3	2 - 4	2	2	2	2
**PD-L1**	**Any**	**Any**				**Any**
**Line**	**Any**	**Any**				**Any**
**N**	29	84				60
**ORR, %**	17.9 %	Not reported				27 %
**DCR, %**	53.6 %	Not reported				37 %
**Median PFS, mo (95% CI)**	2.3 (1.8 – 4.8)	1.7 (1.3 – 2.1)				4.4 (3.3 – 9.9)
**Median OS, mo (95% CI)**	4.1 (2.1 – 6.9)	4.5 (2.3 – 6.9)				9.8 (7.1 – 14.6)
**PD-L1**	**Any**	**Any**				**Any**
**Line**	**First**	**First**				**First**
**N**	21	19				24
**ORR, %**	20 %	Not reported				21 %
**DCR, %**	55 %	Not reported				38 %
**Median PFS, mo (95% CI)**	2.7 (1.8 – 7.6)	Not reported				4.3 (1.9 – 13.1)
**Median OS, mo (95% CI)**	4.3 (2.3 – 7.8)	6.7 (Not reported)				7.9 (2.6 – Not reached)
**PD-L1**	**≥ 50%**					**≥ 50%**
**Line**	**Any**					**Any**
**N**	17					15
**ORR, %**	25 %					47 %
**DCR, %**	43.8 %					53 %
**Median PFS, mo (95% CI)**	2.1 (0.5 – 4.9)					12.6 (1.9 – Not reached)
**Median OS, mo (95% CI)**	2.8 (0.9 – 6.9)					14.6 (4.6 – Not reached)
**PD-L1**	**≥ 50%**		**≥ 50%**	**≥ 50%**	**≥ 50%**	
**Line**	**First**		**First**	**First**	**First**	
**N**	12		153	39	56	
**ORR, %**	27.3 %		21 %	25.6 %	45 %	
**DCR, %**	45.5 %		37 %	Not reported	Not reported	
**Median PFS, mo (95% CI)**	2.1 (0.5 – 4.9)		2.4 (1.6 – 2.5)	4.0 (2.07 – 14.04)	2.6 (1.9 – 5.1)	
**Median OS, mo (95% CI)**	2.8 (0.5 – 6.9)		3.0 (2.4 – 3.5)	7.4 (3.78 – Not reached)	7.8 (2.5 – 10.7)	

© 2021 Sehgal K et al. *JAMA Network Open.*

Abbreviations: ECOG PS = Eastern Cooperative Oncology group performance status, DCR = Disease control rate, mo = months, ORR = Overall response rate, OS = Overall survival, N = Number of patients, NSCLC = Non-small cell lung cancer, PD-L1 = Programmed death ligand-1, PFS = Progression-free survival, TPS = Tumor proportion score

© 2021 Sehgal K et al. *JAMA Network Open.*

eTable 4. Clinicopathologic and Treatment Characteristics of Patients With ECOG PS of at Least 2 With or Without Durable Clinical Benefit

		Durable Clinical Benefit (N = 7)	No Durable Clinical Benefit (N = 22)	P value
**ECOG PS**	2, n (%)<br>3, n (%)	6 (85.7)<br>1 (14.3)	19 (86.4)<br>3 (13.6)	1.00
**Age, years**	Median (range)	68 (63 – 87)	72.5 (42 – 86)	0.68
**Sex**	Female, n (%)	3 (42.9)	11 (50)	1.00
**Race**	Caucasian, n (%)<br>African American, n (%)<br>Asian, n (%)	5 (71.4)<br>2 (28.6)<br>0	16 (72.7)<br>4 (18.2)<br>2 (9.1)	0.79
**Body Mass Index**	Median (range)	25.3 (19.5 – 37.3)	24.9 (17.3 – 40.9)	0.58
**Obesity**	Yes, n (%)	3 (42.9)	5 (22.7)	0.36
**Smoking**	Ever, n (%)	7 (100)	18 (81.8)	0.55
**Comorbidities**	Simplified Comorbidity Score, Median (range)	9 (7 – 13)	9 (2 – 14)	0.88
	Hypertension, n (%)<br>Diabetes mellitus, n (%)<br>Cardiovascular, n (%)<br>Respiratory, n (%)<br>Creatinine clearance <60, n (%)<br>Creatinine clearance <30, n (%)<br>VTE, n (%)	3 (42.9)<br>0 (0)<br>3 (42.9)<br>4 (57.1)<br>3 (42.9)<br>0 (0)<br>1 (14.3)	14 (63.6)<br>4 (18.2)<br>6 (27.3)<br>8 (36.4)<br>8 (36.4)<br>1 (4.5)<br>5 (22.7)	0.40<br>0.55<br>0.64<br>0.40<br>1.00<br>1.00<br>1.00
**Histology**	Non-squamous, n (%)<br>Squamous, n (%)<br>Poorly differentiated, n (%)	5 (71.4)<br>2 (28.6)<br>0	14 (63.6)<br>6 (27.3)<br>2 (9.1)	1.00
**Driver mutations/ alterations (N=26 [7/19])**	None, n (%)<br>KRAS, n (%)<br>EGFR, n (%)<br>Others, n (%)<br>- MET, n (%)<br>- BRAF, n (%)	4 (57.1)<br>2 (28.6)<br>1 (14.3)<br>0<br>0<br>0	6 (31.6)<br>10 (52.6)<br>1 (5.3)<br>2 (10.5)<br>1 (5.3)<br>1 (5.3)	0.38
**PD-L1 TPS**	Median (range)<br>≥ 50%, n (%)	40 (1 – 100)<br>3 (42.9)	70 (1 – 90)<br>14 (63.6)	0.30<br>0.40
**Sites of metastases**	Number, Median (range)	2 (1 – 4)	2 (1 – 5)	0.22
	Lung/pleura, n (%)<br>Lymph node, n (%)<br>Brain, n (%)	5 (71.4)<br>3 (42.9)<br>1 (14.3)	12 (54.5)<br>12 (54.5)<br>8 (36.4)	0.67<br>0.68<br>0.38

© 2021 Sehgal K et al. *JAMA Network Open.*

	Bone, n (%)<br>Liver, n (%)<br>Adrenal, n (%)<br>Others, n (%)	3 (42.9)<br>0 (0)<br>1 (14.3)<br>1 (14.3)	13 (59.1)<br>4 (18.2)<br>3 (13.6)<br>7 (31.8)	0.67<br>0.55<br>1.00<br>0.64
**Line of therapy**	First line, n (%)<br>≥ Second line, n (%)	6 (85.7)<br>1 (14.3)	15 (68.2)<br>7 (31.8)	0.64
**WBC (K/uL)**	Median (range)	7.9 (5.4 – 10.2)	8.6 (4.7 – 37.5)	0.40
**ANC (K/uL)**	Median (range)	5.59 (3.38 – 7.52)	6.92 (3.51 – 22.4)	0.25
**Creatinine (mg/dL)**	Median (range)	0.9 (0.4 – 1.8)	0.85 (0.5 – 2.3)	0.74
**Objective response (N=28 [7/21])**	ORR, n (%)<br>• CR, n (%)<br>• PR, n (%)	4 (57.1)<br>0 (0)<br>4 (57.1)	1 (4.8)<br>0 (0)<br>1 (4.8)	0.008
**Immune-related adverse events**	Any grade, n (%)<br>≥ Grade 3, n (%)<br>Steroid use, n (%)	5 (71.4)<br>1 (14.3)<br>1 (14.3)	4 (18.2)<br>4 (18.2)<br>4 (18.2)	0.02<br>1.00<br>1.00

Abbreviations: ANC = Absolute neutrophil count, CAD = Coronary artery disease, CHF = Congestive heart failure, CR = Complete response, ECOG PS = Eastern Cooperative Oncology group performance status, K/uL = x1000/microliter, ORR = Overall response rate, PD-L1 = Programmed death ligand 1, PR = Partial response, TPS = Tumor proportion score, VTE = Venous thromboembolic disease, WBC = White blood cell count, wk = weeks

© 2021 Sehgal K et al. *JAMA Network Open.*

eTable 5. Treatment Outcomes in Patients With ECOG PS 0-1 vs ECOG PS2

		All (N = 70)	ECOG PS 0-1 (N = 45)	ECOG PS =2 (N = 25)	P value
**Objective response (N=67 [43/24])**	ORR, n (%)	16 (23.9)	12 (27.9)	4 (16.7)	0.38
	• CR, n (%)	2 (2.9)	2 (4.6)	0 (0)	
	• PR, n (%)	14 (20.9)	10 (23.3)	4 (16.7)	
**Disease control (N=67 [43/24])**	DCR, n (%)	51 (76.1)	38 (88.4)	13 (54.2)	0.003
	• SD, n (%)	35 (52.2)	26 (60.5)	9 (37.5)	
**Immune-related adverse events**	Any grade, n (%)	32 (45.7)	24 (53.3)	8 (32)	0.09
	≥ Grade 3, n (%)	11 (15.7)	6 (13.3)	5 (20)	0.51
	Steroid use, n (%)	15 (21.4)	10 (22.2)	5 (20)	1.00
	Treatment discontinuation, n (%)	7 (10)	3 (6.7)	4 (16)	0.24
	Death, n (%)	1 (1.4)	1 (2.2)	0 (0)	1.00
**Progression Free Survival**	Median, months (95% CI)	4.9 (3.3 – 7.9)	7.9 (4.6 – 15.4)	2.3 (1.8 – 4.8)	0.003
**Progression Free Survival-2**	Median, months (95% CI)	8.9 (6.6 – 11.9)	11.9 (8.6 – 23.9)	4.1 (2.3 – 6.9)	<0.001
**Overall Survival**	Median, months (95% CI)	12.7 (6.7 – 23.2)	23.2 (14.0 – 35.7)	4.1 (2.3 – 6.9)	<0.001
**Death on home hospice**	Yes, n (%)	26 (37.1)	15 (33.3)	11 (44)	0.35
	No, n (%)	14 (20)	7 (15.6)	7 (28)	
	Not known, n (%)	2 (2.9)	0 (0)	2 (8)	
	Not applicable, n (%)	28 (40)	23 (51.1)	5 (20)	

Abbreviations: CR = Complete response, DCR = Disease control rate, ECOG PS = Eastern Cooperative Oncology group performance status, ORR = Overall response rate, PR = Partial response, SD = Stable disease

© 2021 Sehgal K et al. *JAMA Network Open.*

eFigure 1. Univariate Kaplan-Meier Survival Curves for Progression-Free Survival-2 in Patients With Advanced NSCLC and ECOG PS 0-1 vs at Least 2 Treated With Pembrolizumab Monotherapy

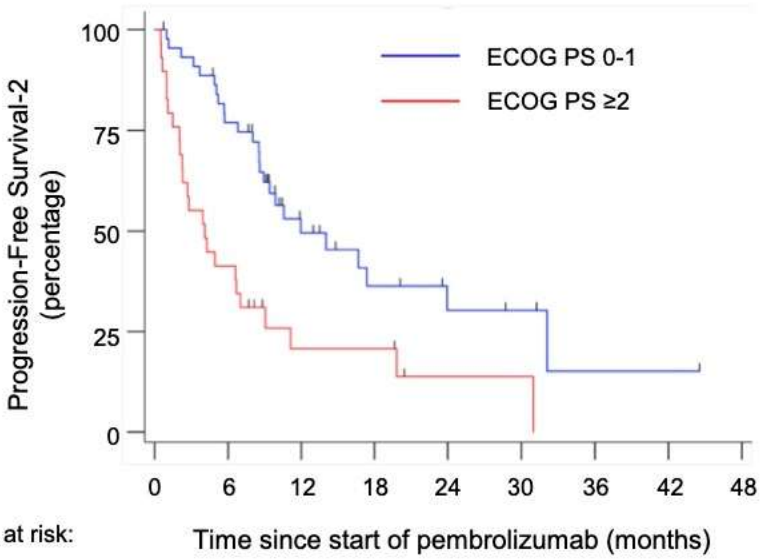

No. at risk:									
PS 0-1	45	33	14	8	5	4	1	1	0
PS ≥2	29	12	4	4	1	1	0	0	0

© 2021 Sehgal K et al. *JAMA Network Open.*

eFigure 2. Univariate Kaplan-Meier Survival Curves for Progression-Free Survival, Overall Survival, and Progression-Free Survival in Patients With Advanced NSCLC and ECOG PS 0-1 vs 2 Treated With Pembrolizumab Monotherapy

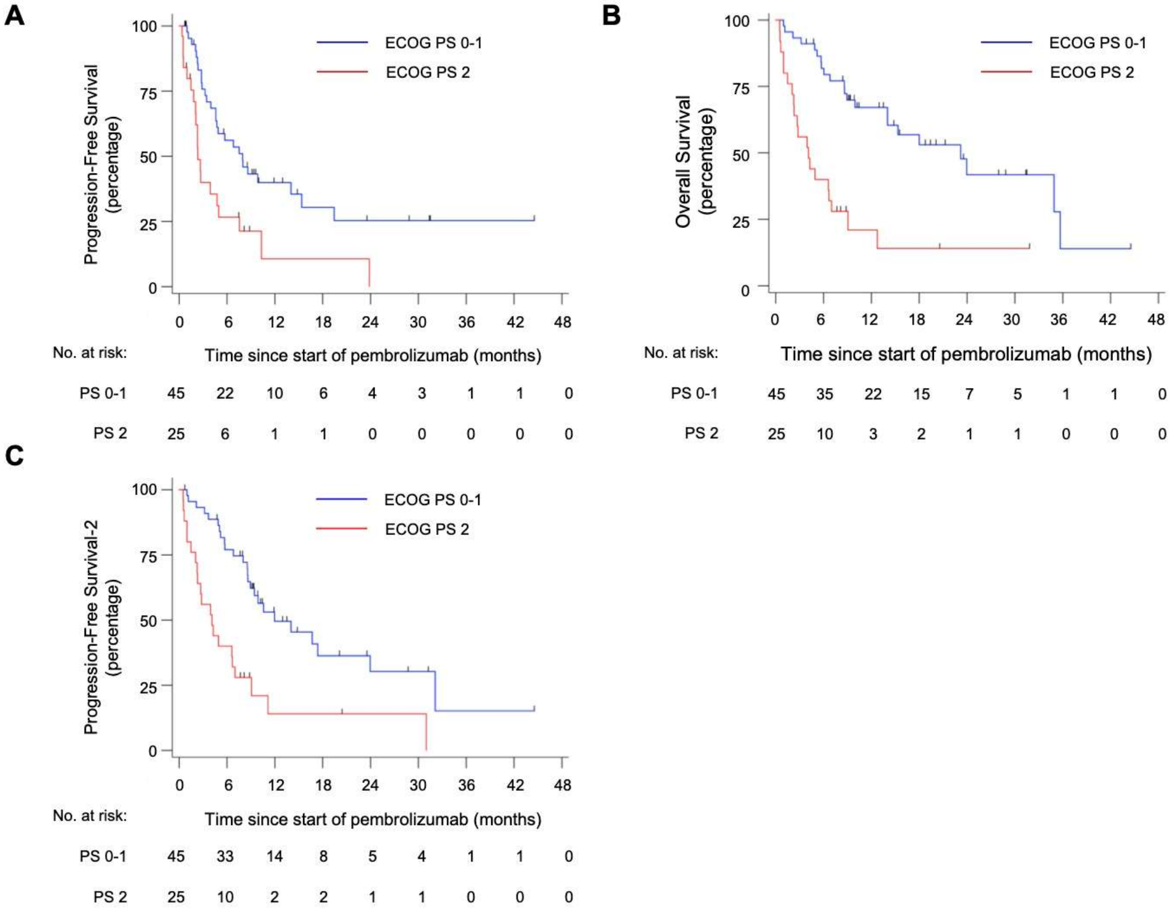

© 2021 Sehgal K et al. *JAMA Network Open.*
